# Relative importance of wildlife and livestock transmission route of brucellosis in southwestern Uganda

**DOI:** 10.1016/j.dib.2018.05.135

**Published:** 2018-05-29

**Authors:** Pius Mbuya Nina, Herwig Leirs, Samuel Mugisha, Patrick Van Damme

**Affiliations:** a Department of Plant Production, Faculty of Bioscience Engineering, Ghent University, Ghent, Belgium; b College of Natural Sciences, Department of Biology, Makerere University, Kampala, Uganda; c Department of Evolutionary Ecology, University of Antwerp, Belgium; d Department of Environmental Sciences and Management, International University of East Africa, Kampala, Uganda; e Faculty of Tropical AgriSciences, Czech University of Life Sciences, Prague, Czech Republic

## Abstract

The data in brief provides a descriptive summary of the field data collected using Eco-health approach in order to support local effort aimed at creating information base for taking evidence-based decisions, especially in regard to wildlife conservation outside protected area and range resource management. The data were collected between June 2012 and July 2014 on a range of issues including wild animals, livestock, household income and cost of diseases control in cattle. In a nutshell the data article shows spatial pattern of a declining brucellosis prevalence in cattle linked to animal population density with increasing distance away from the Lake Mburo National Park (LMNP) boundary in southwestern Uganda. It is the trend of animal distribution in private land that the pastoralist communities perceived as influencing economic losses associated with diseases affecting cattle production. The pastoralists strongly believe that wild ungulates grazing with cattle outside the park on a daily basis present a potential risk of disease transmission which adversely affects their cherished source of livelihood. This article refers to “Brucellosis in cattle and micro-scale spatial variability of pastoral household income from dairy production in south western Uganda. Acta tropica”, Acta Tropica, 2018.

**Specifications Table**

**Table:** 

Subject area	Agricultural and Biological Sciences
More specific subject area	*Ecohealth approach to disease control at wildlife-livestock nexus*
Type of data	*Tables, text file and a figure*
How data was acquired	*Two data sets were obtained one focusing on serological surveys and another on socio-economics of pastoralist households. The first set of data were collected through serological surveys where blood samples were collected from cattle at household level and analyzed for brucellosis in cattle*[4]*. Another data set contained socio-economic data which were collected through interviews with respondents from randomly selected households. The households were mapped prior to the study using a hand-held GPS receiver for easy identification. Cattle blood sample were from the same homesteads selected for the interviews. We also surveyed wild animals’ distribution outside protected area using established transect lines*[3].
Data format	*Raw, filtered and analyzed*
Experimental factors	*Sera were collected from 1962 cattle between August 2012 and June 2013 from 330 homesteads that were proportionately distributed in samples of 55 across six zones along a distance gradient from LMNP. All blood samples were centrifuged and the sera stored at −80* *°C in the microbiology laboratory of Mbarara University, Mbarara before carrying out screening and subsequent confirmatory tests for brucellosis.*
Experimental features	*An indirect multi-species immunosorbent assay (iELISA) using Brucella S-LPS antigen was developed. Serial testing of the cattle sera for anti-B. abortus antibodies was conducted using the Rose Bengal Plate Test (RBPT)*[1]*, and later confirmed with iELISA. A confirmatory positive sample was one that tested positive for both RBPT and I-ELISA (titers 1:80).*
Data source location	*Kiruhura District of western Uganda*
Data accessibility	*Data are contained within this article*

**Value of the data**

• The data variables indicate unique circumstances of brucellosis transmission in cattle and household income that might inform a monitoring plan for local disease control.
• The data provides information evidencing strong concerns the local communities have regarding the presence of wild species of animals on their private farms and ranches around Lake Mburo National Park in southwestern Uganda.
• Therefore, the data in this article allows other interested researchers access and use of raw facts in different ways that might extend statistical analysis and subsequently lead to a more comprehensive understanding of pastoralists’ development trajectory at the wildlife-livestock nexus.

## 1 Methods and materials

### 1.1 Data

**Fig. 1:**
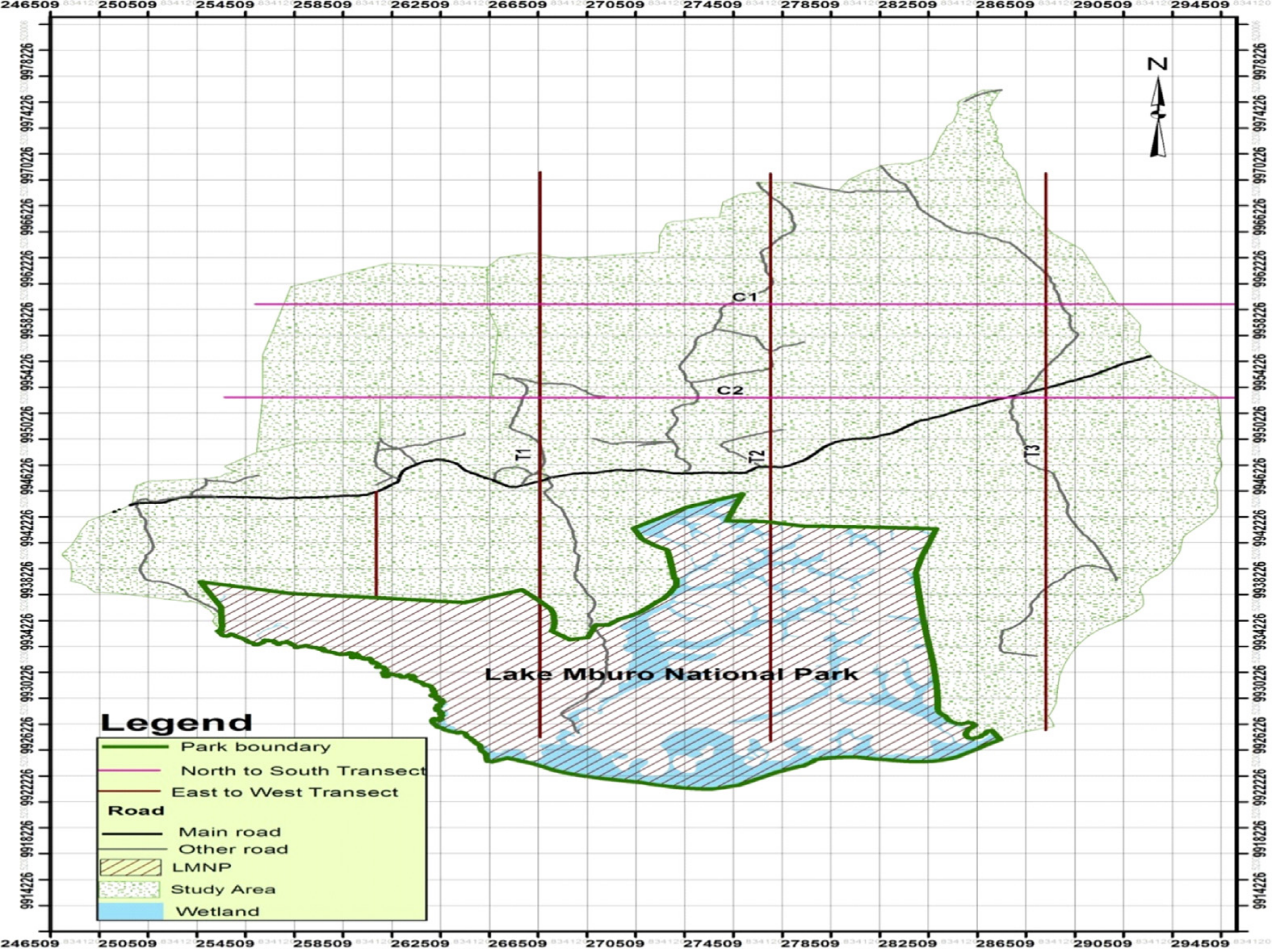
Map of Lake Mburo National Park indicating transect lines laid perpendicular to the northern boundary of LMNP for animal survey.

The dataset in this article contains variables such as spatial pattern of wild animals outside the park, livestock species reared in Lake Mburo conservation area and economic losses pastoralist communities incur due to limitations imposed on cattle production by diseases. The Fig. 1 illustrates the study design adopted for animal surveys during the study that generated the data presented herein. Tables 1–4 show the spatial pattern of wild animals’ distribution and proportions of cattle breeds in each distance zone along a gradient from LMNP. Similarly, Tables 5–7 provide descriptive summary and statistics of major cattle diseases in the study area, brucellosis prevalence in cattle and symptomatic abortions.

**Table 1:** Spatial distribution of cattle breeds along a distance gradient from LMNP boundary.

Dependent variables	Distance from Lake Mburo National Park in Km						
	N	0–4	4–8	8–12	16–20	16–20	20–24
Number of all cattle	26,923	5503	8820	5451	2944	3090	1115
Surviving cattle offspring	6752	1436	1785	1620	730	865	316
Abortions in past month	1900	579	545	467	151	104	35
Indigenous breed of cattle	11,575	2729	3704	2803	1079	891	369
Cross breed of cattle	8651	1947	3566	1717	804	528	89
Exotic breed of cattle	6844	827	1560	951	1053	1779	674
Cattle given veterinary services	3541	717	911	754	433	519	207
Cattle recovered after the treatment	3276	619	860	725	415	473	184

**Table 2:** Descriptive summary of cattle breeds, disease risks, milk production and unit price of milk per household per month.

Parameters	N	Range	Minimum	Maximum	Sum	Mean	Std. error	Std. deviation
Total number of cattle	366	557	0	557	31,110	85.00	4.17	79.71
Surviving offspring	365	80	0	80	7873	21.57	0.72	13.60
Number of abortions in past three months	366	35	0	35	1971	5.39	0.31	5.99
Ankole breed of cattle	366	357	0	357	12,036	32.89	2.47	47.28
Cross breed of cattle	366	433	0	433	9884	27.01	2.33	44.53
Friesian breed of cattle	366	316	0	316	9336	25.51	1.83	34.92
Treated cows in past one month	366	45	0	45	3897	10.65	0.38	7.22
Cows recovered after treatment	366	40	0	40	3567	9.75	0.35	6.71
Milk price in rainy season	366	470	300	500	149,505	408.48	13.19	252.36
Milk price in dry season	366	300	600	900	259,900	710.11	4.81	92.05
Average milk prices	366	452	450	903	204,702	559.30	7.66	146.48

**Table 3:** Average household expenditure on disease control against income from sale of milk in UGX at each distance category from Lake Mburo National Park boundary.

		N	Mean	Std. deviation	Std. error	Mean at 95% CI		Minimum	Maximum
						Lower bound	Upper bound		
Total disease control cost	0 – 4	60	561500.00	370217.87	47794.92	465862.58	657137.42	0.00	1500000.00
	4 – 8	58	718103.45	418127.43	54902.82	608162.44	828044.45	0.00	1440000.00
	8 – 12	64	514921.88	281314.22	35164.28	444651.67	585192.08	85000.00	1160000.00
	12 – 16	60	483750.00	221492.67	28594.58	426532.38	540967.62	0.00	1075000.00
	16 – 20	62	473548.39	207282.15	26324.86	420908.58	526188.21	0.00	960000.00
	20 – 24	62	446048.39	172852.48	21952.29	402152.08	489944.71	0.00	710000.00
	Total	366	530969.95	301886.45	15779.86	499939.10	562000.79	0.00	1500000.00
Milk sales	0 – 4	60	493166.67	309107.47	39905.60	413315.74	573017.59	0.00	1200000.00
	4 – 8	58	621551.72	350827.60	46065.92	529306.31	713797.14	0.00	1300000.00
	8 – 12	64	745390.63	310725.35	38840.67	667773.74	823007.51	90000.00	1500000.00
	12 – 16	60	843000.00	742447.40	95849.55	651205.50	1034800.06	0.00	6000000.00
	16 – 20	62	961612.90	548948.90	69716.58	822205.99	1101000.60	0.00	3000000.00
	20 – 24	62	805645.16	271647.20	34499.23	736659.69	874630.63	0.00	1400000.00
	Total	366	747254.09	475544.29	24857.10	698373.01	796135.21	0.00	6000000.00

**Table 4:** Pooled data on density of wild ungulates and livestock per transect (T) and control (C.) lines collected between June 2012 and March 2013.

**Species of wild animals**	Distance from Park Boundary in Km					
	0–4	4–8	8–12	12–16	16–20	20–24
Zebra - *Equus burchelli* [T]	53.25	75.25	40.5	36.75	18.5	0.5
Zebras - *Equus burchelli* [C]	71.75	10.5	16.25	27.25	4.75	0
Bushbucks - *Tragelaphus scriptus* [T]	17.25	4.75	6	3.5	1.25	1
Bushbucks - *Tragelaphus scriptus* [C]	10.25	2	4	2.75	0.75	0
Impalas - *Aepyceros melampus* [T]	47.75	27.25	25	6.5	1.5	0
Impalas - *Aepyceros melampus* [C]	10.25	96.25	26.75	22.25	2.25	1.75

**Domestic animals grazing in the fields**						
Cows - Mixed Breeds [T]	173.25	320.25	215	253.25	438.5	31.25
Cows - Mixed Breeds [C]	328.75	105.25	188	384	33.75	0
Goats - Mixed Breeds [T]	73.5	71.75	76.5	62.75	63.5	2
Goats - Mixed Breeds [C]	35.25	133.5	66.5	66	61.75	0
Sheep – Local Breed [T]	4.5	6.25	0	17.5	2.75	1.75
Sheep – Local Breed [C]	7.75	6.5	11.25	17.75	0	0

**Note:***Both wild species and domesticated animals sighted along each of the transects were counted and recorded along T = transect lines walked and C = control lines passing across the transect lines.*

**Table 5:** Diseases of great concern to the pastoralist communities around Lake Mburo National Park.

Major diseases	Spatial ranking of cattle diseases ( 0 - 24 km)					
	0 – 4	4 – 8	8 –.12	12.– 16	16 – 20	20 – 24
	(n = 60)	(n = 58)	(n = 64)	(n = 60)	(n = 62)	(n = 62)
Tick & Tick-borne diseases	40(66.7%)	42(72.4%)	47(73.5%)	43(71.7%)	45(72.6)	44(72.6%)
Brucellosis	14(33.3%)	13(22.4%)	13(20.3%)	10(16.7%)	8(12.9%)	9(12.9%)
Foot and mouth	2(3.3%)	2(3.4%)	2(3.1%)	3(5%)	4(6.5%)	4(6.5%)
Others	4(6.7%)	1(1.7%)	2(3.1%)	4(6.6%)	5(8.1%)	5(8.1%)

**Table 6:** Brucellosis sero-prevalence in cattle reared within Lake Mburo Conservation Area.

Distance (km)	Tested	Seropositive	% Prevalence	Sero-positive at household level		
				Minimum	Maximum	Mean ± SE
0–4	292	176	60.27	0	5	3.26 ± 0.12
4–8	291	151	51.89	1	5	2.80 ± 0.11
8–12.	292	152	52.05	1	5	2.81 ± 0.11
12–16	292	114	39.04	0	4	2.11 ± 0.13
16–20	292	94	32.19	0	4	1.74 ± 0.14
20–24	291	79	27.15	0	3	1.45 ± 0.14

**Table 7:** Abortions in cattle perceived by pastoralists as symptomatic effect of zoonotic brucellosis.

Distance in km	H/steads	Abortions per km^2^	Abortions in 100 cattle	
			Mean ± SE (95% CI)	*P*-value
0 – 4	60	144.75	10.52(9.76–11.26)	0.006
4 – 8	58	136.25	8.25(7.45–9.05)	0.014
8 – 12	64	116.75	8.30(7.83–8.77)	0.025
12 – 16	60	45.25	4.64(3.66–5.62)	0.001
16 – 20	62	29.75	2.96(2.04–3.88)	0.001
20 – 24	62	20	2.27(1.75–2.79)	0.001

Note: Selected homesteads within each distance zone were the data collection points.

### 1.2 Experimental design, materials and methods

A population survey of wild ungulates was carried out along 3 transect lines in order to determine any spatial association between location of animals and homesteads from Lake Mburo National Park (LMNP) boundary.

Wild ungulates sighted on livestock grazing farms/ranches along a distance gradient from the LMNP were counted and recorded from June 2012 to July 2014), using a standard method described by Buckland et al. [2] for estimating animal density and abundance. Three transect lines about 8 km apart were laid perpendicular to the northern boundary of LMNP, since wild animals were dispersed to the ranches and farms located on the northern side of the park (Fig. 1).
